# *Zymobacter palmae* Pyruvate Decarboxylase is Less Effective Than That of *Zymomonas mobilis* for Ethanol Production in Metabolically Engineered *Synechocystis* sp. PCC6803

**DOI:** 10.3390/microorganisms7110494

**Published:** 2019-10-27

**Authors:** Lorraine Quinn, Patricia Armshaw, Tewfik Soulimane, Con Sheehan, Michael P. Ryan, J. Tony Pembroke

**Affiliations:** 1 Department of Chemical Sciences, School of Natural Sciences and the Bernal Institute, University of Limerick, V94 T9PX Limerick, Ireland; 2 School of Engineering, University of Limerick, V94 T9PX Limerick, Ireland

**Keywords:** alcohol dehydrogenase (ADH), biofuels, cyanobacteria, ethanol, pyruvate decarboxylase (PDC), renewable energy, *Synechocystis* sp. PCC 6803

## Abstract

To produce bioethanol from model cyanobacteria such as *Synechocystis*, a two gene cassette consisting of genes encoding pyruvate decarboxylase (PDC) and alcohol dehydrogenase (ADH) are required to transform pyruvate first to acetaldehyde and then to ethanol. However the partition of pyruvate to ethanol comes at a cost, a reduction in biomass and pyruvate availability for other metabolic processes. Hence strategies to divert flux to ethanol as a biofuel in *Synechocystis* are of interest. PDC from *Zymobacter palmae* (ZpPDC) has been reported to have a lower Km then the *Zymomonas mobilis* PDC (ZmPDC), which has traditionally been used in metabolic engineering constructs. The *Zppdc* gene was combined with the native *slr1192* alcohol dehydrogenase gene (*adh*A) in an attempt to increase ethanol production in the photoautotrophic cyanobacterium *Synechocystis* sp. PCC 6803 over constructs created with the traditional Zmpdc. Native (Zppdc) and codon optimized (*ZpOpdc*) versions of the ZpPDC were cloned into a construct where *pdc* expression was controlled via the *psb*A2 light inducible promoter from *Synechocystis* sp. PCC 6803. These constructs were transformed into wildtype *Synechocystis* sp. PCC 6803 for expression and ethanol production. Ethanol levels were then compared with identical constructs containing the *Zmpdc*. While strains with the *Zppdc* (UL071) and *ZpOpdc* (UL072) constructs did produce ethanol, levels were lower compared to a control strain (UL070) expressing the pdc from *Zymomonas mobilis*. All constructs demonstrated lower biomass productivity illustrating that the flux from pyruvate to ethanol has a major effect on biomass and ultimately overall biofuel productivity. Thus the utilization of a PDC with a lower Km from *Zymobacter palmae* unusually did not result in enhanced ethanol production in *Synechocystis* sp. PCC 6803.

## 1. Introduction

Recently, much effort has focused on the development of alternative sources of energy that are environmentally friendly and sustainable [1] in comparison to fossil fuels [2,3]. With many alternatives being explored, much research has focused on the metabolic engineering of the model cyanobacterium *Synechocystis* sp. PCC 6803 (referred to hereafter as *Synechocystis* PCC 6803) to produce biofuels such as ethanol [4] from CO_2_ and sunlight photoautotrophically [5]. Metabolic engineering has been utilized to direct *Synechocystis* PCC 6803 to produce a range of products [6] including ethanol via expression of heterologous *pdc* (pyruvate decarboxylase) and *adh*II (alcohol dehydrogenase) genes from *Zymomonas mobilis* (*Zmpdc* and *ZmadhII*).

In the late 1980s, *Escherichia coli* was initially engineered with these genes [7] to produce ethanol as a proof of concept. This was followed by the metabolic engineering of the first cyanobacterium *Synechococcus elongatus* PCC 7942 [8] and soon afterwards *Synechocystis* PCC 6803 was also used to produce ethanol using the same *pdc* and *adhA* genes from *Zymomonas mobilis* using a strong light driven native *psb*A2 promoter. This *Synechocystis* strain gave double the amount of ethanol production compared to the *Synechococcus elongatus* PCC 7942 strain [9]. US biofuel companies Algenol and Joule Unlimited have since worked towards the development of industrial ethanol producing cyanobacteria using an *adh*A (*slr1192*) native to *Synechocystis* PCC 6803 coupled to the *pdc* gene from *Zymomonas mobilis* allowing overexpression of these genes and enhanced ethanol production [10]. To enhance ethanol production further, gene dosage has been used, which employed two copies of the *Zmpdc* and *slr1192 adhA* genes coupled with the knockout of the PHB (poly-β-hydroxybutyrate) storage compound pathway [11] leading to further increased ethanol yields.

Other approaches that hold potential include the use of small native *Synechocystis* PCC 6803 plasmids for expression of the heterologous genes in the ethanol pathway [12], the alteration of pyruvate levels via the over expression or decreased expression of certain enzymes like pyruvate kinase (PK) or phosphoenolpyruvate carboxylase (PPC) [13,14] or the utilization of different promoters [15] for expression of the *pdc* and *adhA* genes [16] to enhance enzyme activity and flux to ethanol. Flux of metabolic intermediates, such as pyruvate, to maintain adequate cell homeostasis is a key issue for all microorganisms. All metabolic engineering strategies for bioethanol production in *Synechocystis* involve a diversion of pyruvate to ethanol [7] and any such diversion occurs at the expense of biomass production [1]. There is thus a balance between the need for biomass and the maximum product yield that is possible [1,7]. To maximize the amount of bioethanol or indeed any other engineered product being able to optimize the kinetics of the first enzyme, in this case pyruvate decarboxylase, to withdraw substrate towards the bioethanol pathway would represent a key strategy towards optimum production.

We hypothesized that the *Zymomonas mobilis pdc* gene could be replaced with a *pdc* gene from *Zymobacter palmae* that possesses a PDC with a reported lower Km value. This could potentially increase flux from pyruvate to ethanol in engineered *Synechocystis* PCC 6803 strains. This possibility was examined via cloning and expression of the *Zppdc* gene, both native (*Zppdc*) and codon optimized (*ZpOpdc*), with these cassettes compared to those expressing the *Zmpdc* with respect to ethanol.

## 2. Materials and Methods

### 2.1. Bacterial Strains

The bacterial strains, plasmids and DNA elements utilized as part of this study are listed in Table 1. *Synechocystis* PCC6803 (glucose tolerant, obtained from K. Hellingwerf, UvA, Amsterdam) cells were maintained at 30 °C on BG-11 medium (Sigma) supplemented with 10 mM TES-NaOH (pH 8.2), 20 mM glucose and 0.3% (w/v) sodium thiosulfate. *Zymobacter palmae* DSM-10491 was obtained from the DSMZ and grown in MY broth (1 g yeast extract, 2 g maltose (20% solution made, filter sterilized, 10 mL added for 2% after autoclaving), 0.2 g KH_2_PO_4_, 0.5 g NaCl). All routine plasmid construction and cloning was performed in *E. coli* using Luria–Bertani (LB) broth. All media were supplemented with appropriate antimicrobial agents as required: ampicillin, 100 μg ml^−1^ and kanamycin, 5–100 μg ml^−1^. All strains were stored at −80 °C in either Luria–Bertani (LB) broth containing 50% glycerol (*E. coli*) or 50% BG-11 medium containing 5% (*v/v*) methanol (*Synechocystis* PCC6803).

**Figure 1:**
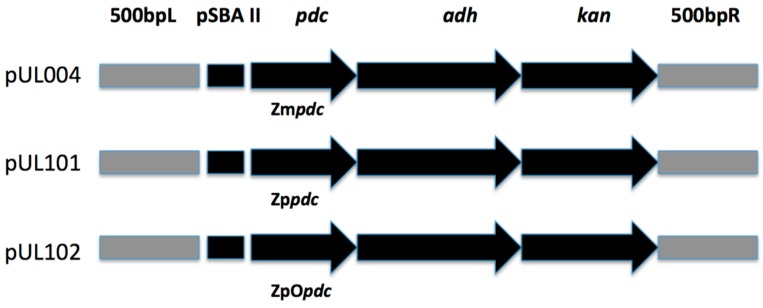
Structure of the recombinant cassettes transformed in *Synechocystis* sp. PCC 6803.

Transformation was carried out via electroporation with electro-competent cells [17]. The gene sequence for *Zppdc* was sent to IDT (Integrated DNA Technologies) to be codon optimized for *Synechocystis* PCC6803. Cassettes were constructed, which contained the *psb*A2 light driven promoter (from plasmid pUL004, Table 1) fused to the *Zmpdc*, the *Zppdc,* and the *ZpOpdc* genes coupled to the native *Synechocystis* PCC 6803 slr1192 *adh*A gene and the kanamycin resistance gene derived from the ICE R391 [18] as described in Lopez et al. [19] (Figure 1). The construct also contained 500 bp at each end with homology to the *psbA2* neutral site to allow homologous recombination into this neutral site [10]. Constructs were generated by PCR amplification of the relevant genes and promoter with fusion of the genes carried out via a biobrick to form the ethanol cassettes similar in structure as previously reported [9,19]. Verification of the pUL101 and pUL102 plasmids was carried out via PCR amplification of the construct (Table 2) followed by sequencing. PCR mixes, Taq polymerase and restriction enzymes for cloning and PCR methods were purchased from Sigma Aldrich. The PCR cycle was as follows: after an initial denaturation at 98 °C for 1 min, 30 cycles of denaturing at 98 °C for 10 s, annealing at 50 °C for 20 s, and extension at 72 °C for 1 min (30 s/kb for ~2 kb gene) extension were undertaken, with a final step at 72 °C for 5 min. PCR products were then analyzed by electrophoresis on a 1.0% agarose gel stained with Sybersafe using 1× TAE buffer as running buffer.

### 2.2. Gene Cloning and Strain Construction

Cloning via homologous recombination was carried out using the In-Fusion^®^ HD cloning kit (Clontech Laboratories Inc.). Primers used can be seen in Table 2.

Transformants of wild type *Synechocystis* PCC6803 were sub-cultured in BG-11 medium containing increasing concentrations of kanamycin (5–100 µg.mL^−1^) until full integration of the cassette was verified. Transformations were left at 30 °C for 16 h under medium intensity white-light illumination (~20–40 µE m^−2^ s^−1^). Verification of integration into the *psbA2* neutral site was carried out with appropriate primers (Table 2) that bound the flanking homologous insertion site within *psbA2*. Wildtype *Synechocystis* PCC6803 amplified with these primers generated a PCR product approximately 1.2 kb in size, insertion of the UL071 and UL072 cassettes resulted in the amplification of a ~4 kb PCR product.

### 2.3. Growth Measurements

Optical density measurements were taken using a Cary UV-Vis spectrophotometer at either 600 nm for *E. coli* or 730 nm for *Synechocystis* sp. PCC 6803 as a measure of biomass yield.

### 2.4. Ethanol Determination

Ethanol determination was carried out using the Yellow line kit: UV-method from R-Biopharm AG. Here, ethanol is oxidized to acetaldehyde via alcohol dehydrogenase (ADH), which in the presence of aldehyde dehydrogenase (Al-DH) is oxidized to acetic acid while NAD+ is reduced to NADH, which was measured at 340 nm via a Cary UV-Vis spectrophotometer. All tests were carried in quintuplicate.

### 2.5. Assay for PDC from Crude Recombinant Extracts of Engineered Synechocystis sp PCC6803

Recombinant strains were adjusted to optical density (OD)_730nm_ of 1.0 and disrupted by beads in pre-chilled buffer [14]. Cell debris was removed by centrifugation. PDC assays on crude preparations carried out as described [20] using sodium pyruvate as substrate and extracts normalized to 1.9 mg.mL^−1^ of protein for assay comparison. Assays were performed in triplicate.

### 2.6. Assays for Acetaldehyde in Recombinant Strains of Synechocystis sp PCC6803

Of the culture 50 mL of each recombinant strain adjusted to equal OD_730nm_ was centrifuged at 15000 g at 4 °C and the cell pellets immediately frozen in liquid nitrogen and extracted as described [21]. Assays for cellular acetaldehyde utilized the EnzyFluo™ Acetaldehyde Assay Kit, BioAssay Systems based on colorimetric acetaldehyde assay coupled to formazan reduction measured at 565 nm. Assays were performed in triplicate.

## 3. Results and Discussion

Bacterial PDCs are rare with only a small number reported. Raj et al. reported four bacterial PDCs from *Zymomonas mobilis* (Zm), *Zymobacter palmae* (Zp), *Acetobacter pasteurianus* (Ap), and *Sarcina ventriculi* (Sv) [22]. By aligning the amino acid sequences of these PDCs, which are similar in length and size (between 552 and 568 amino acids and 59.83 and 61.8 KDa) it was found that the PDC from *Z. palmae* (ZpPDC) shared 72% identity with the PDC from *Acetobacter pasteurianus* (ApPDC) but only 62%/63% identity to the ZmPDC (Table 3) [22,23]. Comparison of kinetic parameters, which can be seen in Table 3, indicated that the ZpPDC might have some potential in metabolic engineering due to its lower Km value in comparison to the ZmPDC. Two other PDCs have since been reported from *Gluconacetobacter diazotrophicus*, GdPDC, and *Gluconobacter oxydans*, GoPDC [24] and are compared in Table 3.

pUL004 (containing *Zmpdc* and described previously [19], pUL101 (*Zppdc*) and pUL102 (*ZpOpdc*; Table 1) were transformed into wildtype *Synechocystis* PCC 6803 to create strains UL070 (pUL004), UL071 (pUL101), and UL072 (pUL102) respectively. The *Zppdc* gene sequence was codon optimized to minimize any possible effect of codon bias in limiting expression in the heterologous host giving UL072. Full segregation of the constructs into the chromosome at the *psbA2* neutral site was confirmed via PCR screening (Supplementary Material). Using primers that spanned the *psbA2* insertion site, wild type strains or those that failed to integrate cassettes into the polyploid *Synechocystis* PCC6803 chromosome resulted in an amplicon of ~1.1 kb. Fully segregated strains that integrated the cassettes also showed one band but at a size of ~4 kb (containing the *psbA2* light promoter, *pdc, adhA,* and kanamycin genes). Strains that displayed partial integration into only some of the polyploid chromosomes showed two bands of both 1.1 and 4 kb (Supplementary Material).

**Figure 2:**
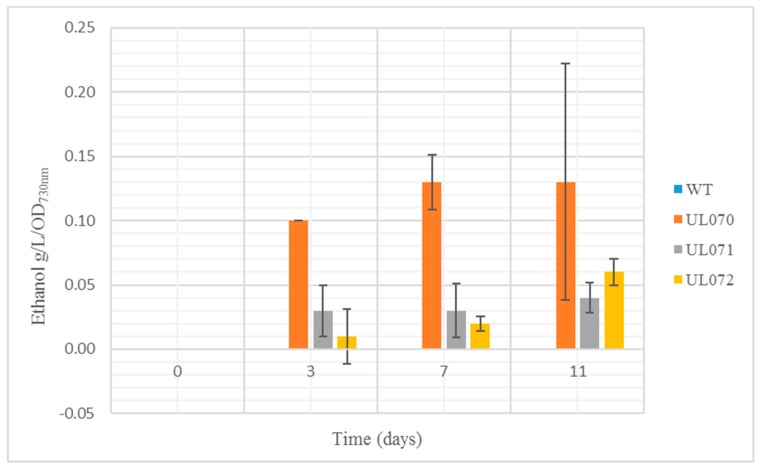
Ethanol levels in g/L/optical density (OD) 730 nm for tested WT(wild type) (Was zero in all cases), UL070 (ZmPDC), UL071 (ZpPDC) and UL072 (ZpOPDC) strains on days 0, 3, 7, and 11 (*n* = 5).

It was decided to test overall ethanol levels to determine construct efficiency, as increasing levels of ethanol were the desired outcome of the research. All strains produced 0 g/L/OD of ethanol on day 0 and levels for each strain subsequently varied over the course of the 3, 7, and 11 days upon growth in BG11 medium. UL070 containing *Zmpdc* produced the largest amount of ethanol at each measurement time in comparison to the other strains (Figure 2).

**Figure 3:**
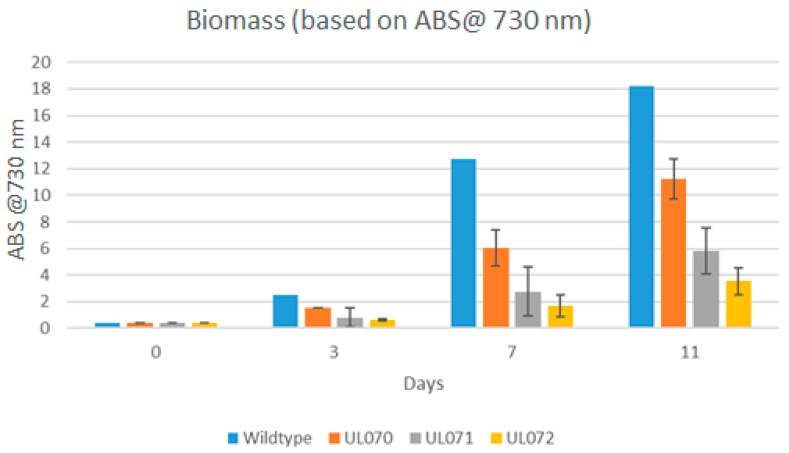
Biomass levels (OD 730 nm) for tested WT, UL070 (*pZmpdc*), UL071 (*pZppdc*), and UL072 (*ZpOpdc*) strains on days 0, 3, 7, and 11 (*n* = 5).

Ethanol levels for the UL071 and UL072 strains were less than the *Zmpdc* expressing strain, which was somewhat surprising, given the lower Km that has been reported for this PDC (we subsequently purified the ZpPDC and verified its reported lower Km, data not shown) [22]. All recombinant strains grew at a slower rate than wildtype that is typical of strains diverting key metabolic intermediates such as pyruvate away from biomass and other metabolic needs. This can be observed in differential optical density (OD) in ethanol producers relative to wildtype strains. All three strains examined showed reduced OD after 3, 7, and 11 days of culture relative to the wildtype *Synechocystis* PCC 6803 strain with UL070 showing the lowest OD, which is an indication that it was most effected by the ethanol production relative to biomass (Figure 3). It can be seen in all cases that the levels of biomass were lower in the three constructs then in the wildtype strain. The level of biomass was also lower in UL071 and UL072 than in UL070.

Liu et al. also used the *Zppdc* gene in a study to increase ethanol production in lactic acid bacteria [28]. The *Zp*PDC was chosen for its low Km for pyruvate and high specific activity amongst all the bacterial PDCs [29]. By using acid inducible and highly conserved constitutive promoters with the *Zppdc*, Liu et al. reported that the acetaldehyde levels produced by the recombinant strains of *Lactococcus lactis* were eight-fold higher in comparison to the control strain but that there was no significant increase in ethanol levels [26,28]. The enzyme has also been recombinantly produced, purified, and structural studies initiated, which may cast more light on its potential [30].

There may be several reasons why the ZpPDC enzyme is apparently less effective than might be hypothesized from its kinetic data. Using cell free extracts from the three recombinant strains UL070, UL071, and UL072 we confirmed that all PDC activities were active in the recombinant strains. It is possible that ZpPDC is converting pyruvate at a faster rate to acetaldehyde compared to the ZmPDC and that this is not coupled effectively to the heterologous ADH resulting in a build-up of acetaldehyde that is known to cause cell toxicity [26]. Attempts were undertaken to measure the acetaldehyde levels (as described in Materials and Methods) however levels were below the detectable limit of the assay kit used. Measurement of acetaldehyde from crude extracts of all three recombinant strains did not identify any detectable alteration in acetaldehyde levels at least under the assay conditions utilized. However measurement of instantaneous acetaldehyde levels in biological systems is difficult with many pitfalls identified ranging from sampling, equilibrium levels, rapid oxidation, volatility, bound forms to various biomolecules, low residual levels, and artifactual acetaldehyde levels [31]. These issues may be factors in being unable to detect variation in acetaldehyde levels in the recombinant strains analyzed. However the lower levels of biomass seen in Figure 3 is suggestive of potential toxicity in the absence of extra ethanol production. To test the possibility that some deficiency in *Adh*A levels might be an issue we transformed pUL101 into a strain termed UL059, which contained the *Zymomonas mobilis adhII* gene under the control of the highly expressed p_trc_ promoter. Ethanol levels in this strain were identical to that of the UL071 strain (data not shown). This data suggested that there did not appear to be a deficiency in ADH for coupling under the experimental conditions used.

Another possibility for the lower than expected ethanol levels recovered from UL071 and UL072 might be the availability of pyruvate as more rapid flux from pyruvate to ethanol would lead to a reduction in biomass as observed. The rationale for using a more efficient PDC would be to avoid the necessity for gene dosage using two copies of the *Zmpdc* gene [11,19], which can lead to gene instability and the need for a double insertion of the cassette. The biomass reduction observed in double cassette strains [11,19] is more obvious than that observed with UL071 or UL072, which suggests that pyruvate availability may not be the limitation in this case. However providing sufficient pyruvate is a key issue in any drive for ethanol and indeed may be addressed by overexpressing pyruvate kinase as has previously been reported [14].

It may also be possible that there is a pH incompatibility issue as the optimum pH for ZpPDC is pH 6.0 [20], which is slightly more acidic than the optimum pH for *Synechocystis* PCC 6803, which is 8.2 with growth showing little reduction up to pH 10 [26]. We examined this possibility by examining crude extracts from all three recombinant strains buffered to pH 6 and pH 8.2 and noted that at the extract pH of pH 8.2 that the residual level of ZpPDC was in fact lower displaying only 60% of the activity observed when the cell extract was buffered to pH 6.0. The ZmPDC demonstrated no apparent loss of activity on dropping the pH to 6.0. We believed this might be at the heart of the issue and that the ZpPDC was not performing optimally in the recombinant strains UL071 and UL072 compared to the ZmPDC in UL070.

In addition to this, the ZpPDC has received little study and so it is possible that there are cofactor differences, metabolic regulatory issues or coupling issues, which have yet to be recognized. Although utilizing a PDC with a lower Km has potential, our data indicates that before such potential can be realized more detailed studies on candidate PDCs will be necessary before progress in this area can be achieved. Should a construct result in higher production levels than this is seen as a success. However if the strategy does not result in greater yields then there are a multitude of potential factors ranging from expression levels, substrate supply, cell pH compatibility, effective coupling with other enzymes in the inserted pathway, and potentially co factor supply and utilization that may be at the root of the issue. Such consequences are diverse and need to be considered when designing experiments for the optimization of metabolic strategies. Thus the utilization of a PDC with a lower Km from *Zymobacter palmae* in both the native and codon optimized form in a pathway for ethanol formation in *Synechocystis* PCC 6803 did not result in an increase of ethanol production levels under the conditions tested with the most likely candidate in this case being intercellular pH incompatibility of the expressed ZpPDC.

## Figures and Tables

**Table 1:** Bacterial strains and plasmids used in this study.

**Strain**	**Genotype/phenotype**	**Source**
BL21 (DE3) *	*E. coli* B F^–^ *ompT, gal, dcm, lon, hsdS_B_*(*r_B_*^–^*m_B_*^–^), λ(DE3 [*lacI lacUV5*-*T7p07 ind1, sam7, nin5*]) [*malB*^+^]_K-12_(λ^S^)	Thermo Fisher Scientific Ballycoolin, Dublin 15, Ireland
DSM-10491	*Zymobacter palmae*	DSMZ—German Collection of Microorganisms and Cell Cultures GmbH
AA314	Wild-type *Synechocystis* PCC6803 strain	K. Hellingwerf, UvA, Amsterdam, The Netherlands
UL004	PCC6803 transconjugant with ZmPDC, *slr1192- adhA, psbA2* locus, *Kan^R^*	[19]
UL059	PCC6803 transconjugant with *Zymomonas mobilis adh*, *ptrc* promoter	This study
UL070	PCC6803 transconjugant with ZmPDC, *slr1192- adhA, psb*A2 locus, *Kan^R^*	This study
UL071	PCC6803 transconjugant with ZpPDC, *slr1192- adhA, psbA2* locus, *Kan^R^*	This study
UL072	PCC6803 transconjugant with ZpOpdc, *slr1192- adhA, psbA2* locus, *Kan^R^*	This study
**Plasmid**	**Genotype/phenotype**	**Source**
pUC18	Amp^R^ backbone plasmid	Sigma-Aldrich, Arklow, Wicklow, Ireland
pUL004	pUC18 backbone, P*psbA2* promoter, *ZmPDC*, *slr1192- adhA, psbA2* integration site, *Kan^R^*	[19]
pUL101	pUC18 backbone P*psbA2* promoter, *ZpPDC*, *slr1192- adhA, psbA2* integration site, *Kan^R^*	This study
pUL102	pUC18 backbone P*psbA2* promoter, *ZpOpdc*, *slr1192- adhA, psbA2* integration site, *Kan^R^*	This study

**Table 2:** Primers used in this study.

Primers	Sequence (5′–3′)
***Zymobacter palmae pdc* primers for the psbA2 vector to create pUL101**	
ZppdcF1	AGGAATTATAACCATATGTATACCGTTGGTATGTACTTGG
ZppdcR1	GATCCCCAAAAACTACGCTTGTGGTTTGCGAGAGTTGG
**Codon optimized *Zymobacter palmae pdc* for the psbA2 vector to create pUL101**	
coZppdcF	AGGAATTATAACCATATGTATACCGTTGGTATGTATTTGG
coZppdcR	GATCCCCAAAAACTATGCCTGGGGCTTCCGGGAATTGG
**Linearize the psbA2 vector to create pUL101 and pUL102**	
PSBAII F	TAGTTTTTGGGGATCAATTC
PSBAII R	ATGGTTATAATTCCTTATGTATTTG
**Sequencing and screening primers for the ethanol cassette (*psbA2* promoter, *pdc*, slr1192 *adhA*, *kan*) in the psbA2 vector**	
P9F	GTCAGTTCCAATCTGAACATCGA
P35F	CTCTACACAGCCCAGAACTATGG
P13R	CAATTTGCAGATTATTCAGTTGGCAT

**Table 3:** Characteristics of known bacterial pyruvate decarboxylases.

PDC	ZmPDC	ZpPDC	ApPDC	SvPDC	GoPDC	GdPDC
**PDB entry**	1ZPD	5EUJ	2VBI	N/A	N/A	4COK
**Bacterial species**	*Zymomonas mobilis*	*Zymobacter palmae*	*Acetobacter pasteurianus*	*Sarcina ventriculi*	*Gluconobacter oxydans*	*Gluconacetobacter diazotrophicus*
**Gram**	Negative	Negative	Negative	Positive	Negative	Negative
**Gene**	M15393	AF474145	AF368435	AAL18557	KF650839	KJ746104
**Protein**	AAA27696	AAM49566	AAM21208	AF354297	AHB37781	AIG13066
**Amino acid identity %**	*62/63	Reference	73	31	67	71
**Kinetics**	* M–M	* M–M	* M–M	* Sigmoidal	* M–M	* M–M
**Km mM (pH)**	* 0.43(6.0) 0.94 (7.0)	* 0.24 (6.0) 0.71 (7.0)	* 0.39 (5.0) 5.10 (7.0)	* 5.7 (6.5) 4.0 (7.0)	# 0.12 (5.0) 1.20 (6.5) 2.80 (7.0)	# 0.06 (5.0) 0.60 (6.0) 1.20 (7.0)
**Optimum Temperature °C**	* 60	* 55	* 65	N/A	#53	# 45–50
**Optimum pH**	* 6.0	* 5.5–6.0	* 5.0–5.5	* 6.3–6.7	# 4.5–5.0	# 5.0–5.5
**Reference**	[24,25]	[23]	[26]	[22]	[27]	[24]

M–M: Michaelis–Menten.

## Supplementary Materials

The following are available online at https://www.mdpi.com/2076-2607/7/11/494/s1: Figure S1: pUL101, Figure S2: DNA agarose gel of PCR amplicons. Table S1: Ethanol yields as measured in g/L/OD for wild-type and metabolically engineered strains (*n* = 5).
